# The Stroop Effect Occurs at Multiple Points Along a Cascade of Control: Evidence From Cognitive Neuroscience Approaches

**DOI:** 10.3389/fpsyg.2019.02164

**Published:** 2019-10-09

**Authors:** Marie T. Banich

**Affiliations:** Institute of Cognitive Science, Department of Psychology and Neuroscience, University of Colorado Boulder, Boulder, CO, United States

**Keywords:** Stroop, fMRI, dorsolateral prefrontal cortex, anterior cingulate, event-related potential

## Abstract

This article argues that the Stroop effect can be generated at a variety of stages from stimulus input to response selection. As such, there are multiple loci at which the Stroop effect occurs. Evidence for this viewpoint is provided by a review of neuroimaging studies that were specifically designed to isolate levels of interference in the Stroop task and the underlying neural systems that work to control the effects of interference at those levels. In particular, the evidence suggests that lateral prefrontal regions work to bias processing toward the task-relevant dimension of a Stroop stimulus (e.g., its color) and away from the task-irrelevant dimension (e.g., the meaning of the word). Medial prefrontal regions, in contrast, tend to be more involved in response-related and late-stage aspects of control. Importantly, it is argued that this control occurs in a cascade-like manner, such that the degree of control that is exerted at earlier stages influences the degree of control that needs to be exerted at later stages. As such, the degree of behavioral interference that is observed is the culmination of processing in specific brain regions as well as their interaction.

## Introduction

The premise of this article is that neuroimaging studies can provide unique insights into the locus of the Stroop effect. For purposes of this paper, we will define the Stroop effect as the inference that occurs between two dimensions of stimulus, one of which is task-relevant and one of which is task-irrelevant. Generally, when these two dimensions are incongruent (e.g., the word “red” printed in blue ink), more cognitive control is required than when the task-irrelevant information is congruent (e.g., the word “red” in red ink) or has no relationship to the task-relevant information (e.g., the word “sum” in red ink). In this paper, it will be argued that this interference can occur at a variety of levels. Furthermore, I will argue that neuroimaging studies can help identify the loci at which such interference occurs to a degree that may not always be possible in behavioral studies.

More specifically, behavioral studies have limitations in isolating the locus of the Stroop effect because it reflects the sum of processes yielding a final outcome of processing as reflected in reaction time or error rates. Since, as will be argued, the Stroop effect can be generated, and also influenced by control, at multiple levels along a cascade of control, cognitive neuroscience approaches can help to identify the multiple levels of interference and control. While careful experimental design can help to elucidate some of these loci, neuroimaging can provide insights into the potential loci of the Stroop effect even when behavioral differences between conditions or individuals are minimal or non-existent. This situation arises exactly because of the cascading nature of control, such that certain brain regions may be able to compensate or “pick up the slack” for reduced or ineffective control at earlier stages in the cascade.

Yet, at the same time, simply examining which regions of the brain become active during performance of the Stroop task is not likely to yield critical information with regard to the potential loci of the Stroop effect. While there have been a number of meta-analyses to isolate brain regions consistently engaged during performance of Stroop-related paradigms with regard to both the more traditional Stroop tasks (e.g., Derrfuss et al., 2005) and variants (Feng et al., 2018), they do not necessarily provide insight into the locus of the Stroop effect. The reasons are that such meta-analyses aggregate findings across different variants of the Stroop task (discussed in more detail below) that may differ in the specific locus or loci that are most engaged by that variant (e.g., a vocal response vs. manual response Stroop task). Furthermore, such studies are often designed to examine cognitive control in general and not specifically designed to uncover the potential loci of the Stroop effect.

**FIGURE 1:**
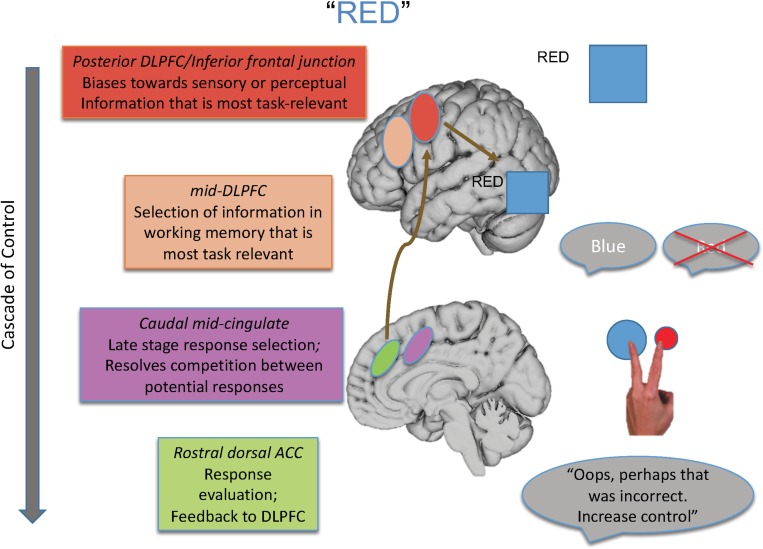
Shown here is the cascade-of-control model outlining the brain regions that are involved in controlling interference in the Stroop task. For an incongruent trial, such as the word “red” in blue ink, control is implemented via a cascade. First posterior regions of DLPFC bias toward task-relevant information relative to task-irrelevant information, as shown by the larger representation of the color blue than the word red. Next mid-DLPFC regions bias toward the relevant information to be maintained in working memory, here biasing toward maintaining the representation of blue and not red. At the next point in the cascade, posterior portions of ACC bias toward the response linked to blue and not toward that linked to red, depicted by the larger blue circle. Finally, more rostral regions of ACC are involved in response evaluation and sending information to DLPFC to adjust control. Importantly, the degree to which one region is active in controlling Stroop interference depends on how well control has been implemented at prior points in the cascade.

For that reason, in this paper, I review the findings of studies designed to isolate the different loci of the Stroop effect and their neural underpinnings, many of which are drawn from our laboratory’s program of research that has melded specific behavioral paradigms with a cognitive neuroscience approach. From such work, we have proposed a model elucidating the brain systems that act with regard to the various loci of interference that can be engendered during the Stroop task (see Figure 1), as well as outlining a cascade of control between brain regions that influences the final behavioral interference effect that is observed.

As an overview, the cascade-of-control model suggests there are at least four important processes and brain loci that influence the Stroop effect. The first process, implemented by posterior regions of lateral prefrontal cortex, biases processing in posterior brain regions toward information that is most task-relevant and/or away from information that is task-irrelevant. The second process, implemented by mid-dorsolateral regions, biases selection toward that information in working memory that is most relevant for the current task goal. The third process, implemented by caudal mid-cingulate regions, is involved in late-stage selection, usually those that are response-related. Finally, rostral dorsal regions of the anterior cingulate cortex (ACC) evaluate the appropriateness of the response selected and send feedback to lateral prefrontal regions to make adjustments in control as needed.

Importantly, this model argues that the degree of Stroop interference observed and how it is controlled depend on how well earlier portions of the cascade, in this case mediated by lateral prefrontal regions, create an appropriate task set. To the degree that such control is not well enabled, medial brain regions, most notably portions of the ACC, must then exert control at later response-related stages of selection. Hence, the “locus” of the Stroop effect in any given experiment is influenced by the activity in and relationship between brain regions, as well as by the specific attributes of a given Stroop paradigm with regard to how much it taxes each of the four processes described above.

Before turning to the studies supporting this model, it should be noted that for purposes of this paper, the classic Stroop task as well as variants will be considered. Because what people describe as a “Stroop task” actually encompasses a family of tasks, we use a specific-naming convention to provide a bit more precision regarding the tasks being discussed. The phrase before the hyphen refers to the task-relevant dimension and the phrase after the hyphen refers to the task-irrelevant dimension. So, for example, the classic Stroop paradigm will be referred to as the color-word Stroop task, as the individual must identify the color in which an item is presented and ignore the meaning of the word.

## Interference Between Two Processes that Vary in their Automaticity or Control Demands

One level at which the Stroop effect occurs is through competition between two distinct processes, one that is more automatic and engaged by the task-irrelevant dimension and another that is less automatic, but which requires processing of the task-relevant dimension so as to meet task demands. In the classic color-word Stroop paradigm, word reading is more automatic than color identification. As such, it requires cognitive control to overcome the tendency to read the word and base a decision on that information to prioritize processing of ink color so as to guide responding.

This aspect of the Stroop task is well captured in computational models of the Stroop task, which includes a “prefrontal” unit that increases activation in units processing color so as to bias the competition toward that process, rather than word identification, in influencing response selection (Cohen et al., 1990). Behavioral evidence suggests that indeed it is the degree of engagement of the word reading process that influences the size of the Stroop effect (Monsell et al., 2001), with greater increases in the latency of color naming for words and pseudowords, which are more likely to engage word reading processes, than for consonant strings, “XXXXXs” or false fonts, which are less likely to engage word-reading processes.

Sometimes, word reading can be engendered not because of the “word-likeness” of letter strings, but because the meaning of the words enable attentional capture. This likely is the locus of interference observed in the emotional Stroop task, which is in essence a color-emotional word task by our nomenclature. In this task, there are various conditions. In one condition, the color of emotionally salient words, which can be either negative or positive in valence, such as “murder” or “joy,” must be identified as compared to emotionally neutral words (e.g., “bench”). Here, word reading is engaged for emotionally salient words because they are thought to capture attention as compared to emotionally neutral words, making identification of the ink color difficult. In fact, the interference effect is reduced in this task vis a vis interference on the color-word task (e.g., Kaiser et al., 2015). Moreover, effects in the emotional Stroop task are sometimes hard to observe and may only occur in those individuals for whom the words have particular emotional significance (e.g., threat words for individuals who suffer from anxiety).

Conversely, manipulations that make the task-relevant dimension more salient can reduce the Stroop effect. In one study, Krebs et al. (2013) used a picture/scene-word Stroop task, in which individuals decided whether a picture represented an indoor or an outdoor scene on which was superimposed a task-irrelevant word (“outside” or “inside” in Dutch). Participants had previewed some of the picture scenes prior to the Stroop task while others were novel. Novel pictures, which are more likely to capture attention, were associated with reduced behavioral interference.

Although one must be careful in making reverse inferences from patterns of brain activation to cognitive processes (Poldrack, 2011), brain imaging studies can provide insights into the degree to which this competition between a more automatic and less automatic process engenders Stroop interference. In a study to examine this issue, Banich et al. (2000b) compared brain activation for two variants of the Stroop task, the standard color-word task and a color-object task, to reveal that automaticity of processing is critical for engaging cognitive control regions, more specifically the dorsolateral prefrontal cortex (DLPFC) and the inferior frontal gyrus (IFG). In the color-object task, individuals had to identify the color in which an object was displayed. On incongruent trials, objects were shown in an atypical color (e.g., a frog displayed in red, when frogs are typically green; a banana displayed in blue, when bananas are typically yellow). Brain activation was compared to neutral trials, in which the object displayed typically can occur in a variety of colors (e.g., a car displayed in red, when cars can be red, blue, gray, white, black, green, etc.).

For both the color-word and color-object tasks, for a given condition, individuals were told to monitor one (but not the other) dimension of the stimuli, making it task-relevant. Their task was to indicate when an item with a given characteristic appeared. For example, for the color-object task, one condition required individuals to monitor for an item in a specific color (e.g., purple), making color task-relevant, while in the other conditions, they monitored for a given “word” (a non-sense word), making the word task-relevant. Likewise, in the color-object task, in one condition, individuals were once again told to monitor for the color purple, and in the other condition, to monitor for a non-sense shape (making shape task-relevant).

Importantly, while color identification is less automatic than word identification in the color-word task, color identification is more automatic than object identification in the color-object task. Hence, if automaticity of processing is indeed a locus of Stroop interference, the pattern of brain activation should be influenced more by the relative automaticity of processes, rather than the nature of the attribute being attended to (i.e., color). Importantly, distinct patterns of brain activation were observed for color monitoring depending on whether it was the less automatic process, as in the color-word task, in which case prefrontal mechanisms were engaged or the more automatic process, as in the color-object task, in which no prefrontal activity was observed. As such, this study provided evidence that relative competition between the automaticity of processes is one locus at which Stroop interference occurs, and that prefrontal regions are involved in control over such effects.

A subsequent study demonstrated that prefrontal mechanisms are engaged when a less automatic process must guide responding, regardless of the specific nature of that process (Banich et al., 2000a). In this study, activation for the contrast of incongruent vs. neutral trials in a color-word Stroop task was compared to that in a spatial-word Stroop task. In the color-word task, incongruent trials consisted of color words displayed in conflicting colors (e.g., “red” in blue ink) while neutral trials consisted of non-color-related words displayed in a particular ink color (e.g., “lot” in blue ink). In the spatial-word task, individuals pressed a button to identify whether a word appeared above, within, or below a box. On incongruent trials, the word’s position conflicted with its meaning (e.g., the word “above” positioned below the box), while on neutral trials, a non-spatial-related word was displayed (e.g., the word “civil” positioned below the box). Overlapping regions of DLPFC were activated for these two tasks, indicating that the need to overcome the automaticity of word reading can be engendered regardless of the nature of the task-relevant attribute (color vs. spatial position).

Taken together, this experiment and the one discussed just above demonstrate that it is not the nature of information in a given stimulus dimension that drives Stroop interference, but rather the relative automaticity of the two processes. In the first study discussed, different patterns of activation were observed when color was the task-relevant dimension, depending on the nature of its automaticity vis a vis the task-irrelevant dimension. In the second study, similar patterns of activation were observed even when the task-relevant attribute differed because processing each of those dimensions was less automatic than word reading.

If indeed it is the automaticity of word reading vis a vis another process that engenders Stroop interference, then one should observe similar patterns of brain activation for the color-word and color-emotional word Stroop task. A direct comparison in the same participants showed that that DLPFC activity is observed for the incongruent condition of a color-word task as well as trials in a color-emotion word task containing either a positive and negative emotionally valenced word compared to a neutral non-emotional word (e.g., “integer”) (Compton et al., 2003). These findings are consistent with the idea that the automaticity of word reading or attentional capture by the word so as to engage word reading must be overcome to enable successful color identification. This overlapping pattern of activation in the frontoparietal network (DLPFC and parietal regions) for the color-emotion word task as compared to the color-word task has been observed in additional non-clinical samples both with positively and negatively valenced words (Kaiser et al., 2015) as well as for positive and threat words (Mackiewicz Seghete et al., 2017).

The effects observed in the color-emotional word task suggest that to the degree that a word is salient, it will capture attention so as to enhance word processing. If so, this should be a general mechanism that can help to increase Stroop interference. This idea is supported by a study (Compton et al., 2003) in which the words were specifically varied in terms of their arousal ratings. More activation was observed in frontoparietal regions for negative words high in arousal as compared to those low in arousal, suggesting that it is the salience of the word that engenders a greater need for control. Demonstrating that this is a general effect not specific to emotion words *per se*, in another study, the frequency with which certain items appears was varied, such that a subset of words occurred less frequently (i.e., oddball trials). DLPFC activation was enhanced for these oddball trials as compared to more frequent trials (Milham et al., 2003a). Thus, any of a number of manipulations that make words more salient so as to increase the engagement of word processing seems to be one locus of the Stroop effect.

## Interference Based on Different Levels of Stimulus-Related Representations

While standard computational models of the Stroop task suggest that it can be explained by competition between two distinct processes, neuroimaging data provide a more complicated picture. In particular, if that were simply the only locus of the Stroop effect, then the identity of the task-irrelevant information should not affect brain activation, as for all intents and purposes it is downregulated relative to the task-relevant dimension.

However, neuroimaging research provided a contrary result. Greater activity was observed in brain regions that process the task-irrelevant attribute for incongruent as compared to neutral trials (Banich et al., 2001), a finding at odds with a simple downregulation of task-irrelevant processing. More specifically, different regions of posterior cortex showed greater activity on the contrast of incongruent (e.g., “red” in blue ink) vs. neutral trials (e.g., “lot” in blue ink) for the color-word task as compared to the contrast of incongruent (e.g., a red frog, when frogs are typically green) vs. a neutral trial (e.g., a red car, when cars can be red among a variety of other colors) in the color-object task. As such, there must be an additional level of competition and/or selection.

In an attempt to understand the factors that drive this pattern of brain activation, Herd et al. (2006) modified the standard computational model of the Stroop task so that it was able to replicate the pattern of brain activation observed as well as the behavioral pattern of results. In the standard computational model, there is an input layer with two subsections – one for the receipt of color information and one for the receipt of word information. These are each linked to an output layer that governs responding. A prefrontal control node modulates processing so as to increase activation of information in the color portion of the input layer in comparison to the word portion of the input layer.

The revised model had three important modifications. First, it included a layer between input and output meant to represent processing of information in posterior cortex in color-specific and word-specific regions, respectively. Part of the goal of including this layer was to see if activation in these portions of the model could mimic the activation observed in posterior brain regions in the empirical neuroimaging studies. Second, it included an additional top-down node to bias toward the abstract concept of color as being critical for the task set. The rationale was that, outside of the Stroop task, individuals typically do not have an abstract representation of color that excludes color words. As such, a task set for “color” is likely to broadly activate information related to the semantic category of color, regardless of whether it is contained in the task-relevant or the task-irrelevant dimension of the Stroop stimulus. Third, also related to the semantics of color, the model was modified so that there were excitatory linkages between representations of color in the ink processing layer (e.g., green) with the related representation in the word processing layer (e.g., “green”).

This model could replicate both the behavioral results of the Stroop task (i.e., longer RT for incongruent than neutral trials) and also patterns of brain activation with more activity in the color processing layer for incongruent than neutral trials. An additional virtue of creating such a model is that portions of it can be “lesioned” to determine what aspect of its architecture is critical to engendering its results. Suggesting that the alterations to the original computational model were critical, neither a model that had the top-down color biasing unit removed nor a model without reciprocal connections between related semantic features could replicate the observed empirical results. Hence, the outcome of this computational modeling suggests that it is the color-relatedness of a representation that serves as a locus of interference.

While the color-relatedness of items is important, studies suggest that the nature of representation to which the semantic category of color is linked can vary and yet still produce interference. Support for this assertion comes from comparison of activation for incongruent vs. neutral trials for three types of Stroop tasks: the standard color-word Stroop task, the color-object Stroop task, and a color-object word Stroop task. As noted above, in the color-object task, an object with a typical color is displayed in an atypical color (e.g., a frog in red) on incongruent trials, while on neutral trials, an object is displayed in one of the many different colors in which it can appear (e.g., a car in red). In the color-object word task, the person simply views the word describing an object that has a typical color (e.g., “frog”), rather than seeing a pictorial depiction of the object. Distinct regions of cortex showed activation depending on the nature of the task-irrelevant attribute, suggesting that it was not just an amodal semantic representation of color that is the source of interference. For example, different regions of the ventral visual processing stream are activated on incongruent trials for the color-word task as compared to the color-object task, suggesting that interference may arise from more orthographically based as compared to visual form-related representations in the former task as compared to the latter. In addition, different portions of the IFG (BA 45 vs. BA 48) became active for the color-object as compared to the color-object word task despite the fact that the interference would arise from the same semantic characteristic (e.g., semantic memory with regard to frogs creates interference because they are typically green not red) (Banich et al., 2001). This finding also suggests that interference can arise at multiple stimulus-related levels.

Another way to examine stimulus-related representations of color is to compare patterns of activation when items have color-related information in both the task-relevant and task-irrelevant dimension as compared to when color-related information is restricted solely to the task-relevant dimension. One can examine this question by determining patterns of brain activation common across both incongruent and congruent trials that are greater than those observed on neutral trials. Investigations taking such an approach (Milham et al., 2002; Milham and Banich, 2005) show that there is not only increased activation in DLPFC, which presumably reflects a more general increased need for control to bias toward task-relevant information, but also increased activation in ventral lateral prefrontal cortex, portions of which are regions involved in semantic retrieval and selection (Badre and Wagner, 2007). Also suggesting interference at the semantic level, left temporal language areas show activation for the contrast of incongruent and congruent trials, which contain semantically related color information in both the ink color and the word, as compared to neutral trials, which in this case were words unrelated to color (e.g., “lot”) (Milham and Banich, 2005).

In sum, the work reviewed in this section suggests that interference can potentially arise in the Stroop task at a number of stimulus-related dimensions, from visual form to orthography, as they relate to the task-relevant category, and also with regard to semantic representations of task-relevant information.

## Response-Related Aspects of Interference

Another series of studies provided evidence that Stroop interference is also engendered at response-related levels. In the first study of this nature, brain activation was examined for two types of incongruent trials, response-eligible and response-ineligible. In response-eligible trials, the competing word also names a potential response. An example would be the word “red” printed in blue ink when the potential responses are red, blue, and green. Response-ineligible trials on the other hand name competing colors, but those that are not a potential response, such as the word “purple” printed in blue ink, when the potential responses are red, blue, and green. If a particular brain region is specifically engaged in dealing with response conflict, it should show greater activation to response-eligible than response-ineligible trials. Importantly, in addition, this region should also show no more activation to response-ineligible trials, which have semantic conflict but no response conflict, than to neutral trials, which have neither semantic nor response conflict (e.g., the word “mile”). A region of mid-cingulate cortex showed such a pattern (Milham et al., 2001), which was confirmed in a subsequent study (Milham et al., 2003a).

Another way to examine response-related aspects of Stroop interference is to compare processing on different blocks of trials in which the stimulus-response mapping is one-to-one as compared to one-to-many. More specifically, on some blocks, each incongruent response-ineligible word was mapped to a different color (e.g., the word “purple” shown in blue, the word “violet” shown in green, etc.), whereas in other blocks, the same task-irrelevant word was presented but paired with a variety of colors (e.g., shown on some trials in blue, in other trials in green, etc.). Hence, stimulus-response mappings were more overlapping in the former condition than the latter. Each of these blocks also contained neutral words (e.g., the word “closet”) with one-to-one as compared to one-to-many color mappings within the appropriate blocks. While DLPFC showed greater activity for incongruent vs. neutral trials, regardless of the nature of the response-mapping (1 to 1; 1 to 4), the ACC was sensitive to the response mapping, showing more activity when the color-response mappings were overlapping (one word to four colors) and hence harder to distinguish than when they were one-to-one (one word to one color) (Liu et al., 2006).

Another way in which response-related interference in the Stroop task has been investigated is via an integrated Simon-Stroop task. In the Simon task, interference arises from stimulus-response interference. In this task, interference is engendered when a right-sided (e.g., right hand) response is required to a left-sided stimulus (and vice versa) as compared to when the location of the item to be responded to and the effector making the response are on the same side of midline. In our integrated Simon-Stroop task, individuals viewed arrows that were located either to the right or left (Simon stimuli), or on different trials above or below (Stroop stimuli) a fixation point. Individuals were trained, for example, to press a right button for an upward arrow and a left button for a downward arrow. Simon interference, which is considered stimulus-response interference, was engendered by placing, for example, an upward arrow to the left of fixation, which then required a right button response to a left-sided stimulus. Stroop interference, which is considered engendered by conflict between two stimulus dimensions, occurred for example when an upward arrow was positioned below the fixation point.

While the contrast of incongruent vs. congruent trials yielded activation in DLPFC for both tasks, the Simon task trials generated activity in motor and response-related regions including the ACC and supplementary motor area (SMA), activity that was not observed in this spatial arrow–spatial position Stroop task. In contrast, the stimulus–stimulus interference of the Stroop task engendered activity in inferior parietal and inferior frontal regions that was not observed in the Simon contrast (Liu et al., 2004). Hence, this body of work suggests that another locus of Stroop interference is at response-related aspects of processing. Consistent with this supposition, certain limitations of the classic computational model of the Stroop task by Cohen et al. (1990) with regard to fitting aspects of human performance can be overcome if the model includes a mechanism for performing final response selection (Stafford and Gurney, 2007).

## An Integrative Model: Stroop Interference Can Occur at Multiple Points Along a Cascade-of-Control

The work described above suggests that Stroop interference can occur at multiple levels. How then can one integrate these findings to shed light on the locus of the Stroop effect? We have argued that, importantly, the degree to which control is exerted at one level of processing can then influence the degree to which interference is engendered or controlled at another.

A pair of early studies helped this idea to come into focus. As reviewed above, our work suggests a broad distinction between control engendered at the level of an abstract task set, mainly implemented by lateral prefrontal cortex, as compared to more response-related aspects of control, mainly implemented by medial prefrontal cortex. In examining differences in brain activation common to incongruent and congruent as compared to neutral trials (e.g., the word “lot”) in the color-word task, there was a notable difference in patterns of activation for younger vs. older adults (Milham et al., 2002). In particular, younger adults exhibited more activation across frontal and parietal regions. Such findings are consistent with reported compromise with aging of prefrontal regions and processes involving executive function and cognitive control (Lockhart and DeCarli, 2014). In contrast, older individuals had more activation in portions of the ACC and SMA. This led us to consider the possibility that due to the lack of top-down control, older individuals were potentially utilizing more response-related mechanisms to deal with the interference.

The converse effect was observed in a study of practice-related effects on the Stroop task. Since the Stroop effect can be maintained over tens of thousands of trials due to the automaticity of word reading, a Stroop task was used in which the interference effect could be reduced with practice. In this task, individuals were trained to assign a color-word label to a series of nonsense designs (e.g., nonsense design 1 was labeled “blue”). Then, later, they were shown either incongruent trials, in which a specific nonsense design was displayed in an incongruent color (e.g., nonsense design 1 labeled “blue” shown in yellow), or neutral trials, on which the nonsense designs were shown in white. To examine learning effects, the experiment was divided into thirds, examining activation for the first third, second third, and last third of trials. While lateral prefrontal activity stayed relatively static across the three portions of the task, that of medial prefrontal activity declined, as did the behavioral Stroop effect, suggesting that individuals were gaining better control over interference. We interpreted this pattern as suggesting that less late-stage response-related interference was occurring, as reflected in reduced ACC activity, due to better top-down control by lateral prefrontal regions, which stayed engaged across all portions of the task (Milham et al., 2003b). Thus, ACC activity depends, in part, on the degree of interference control engendered by DLPFC.

Testing the idea that ACC activity depends in part on the degree of prior control exerted by DLPFC required using a method that afforded better temporal resolution than that provided by fMRI. The relationship between activity in DLPFC and ACC was examined by utilizing event-related potentials (ERPs) due to their superior temporal resolution, in conjunction with fMRI. Participants performed the Stroop task in the magnet and then again while electrophysiological recordings were made. fMRI results were used to enable source localization for ERP waveforms for the DLPFC and ACC. The relationship between ERPs generated by these sources was examined, in addition to how well they could predict, as tested via mediation models, interference on the Stroop task (indexed by the difference in performance between incongruent and congruent trials). The specific model examined whether the influence of DLPFC activity in the 300–440 ms time range on Stroop performance would be mediated, in part, by later ACC activity in the 520–680 ms time range. This pathway was significant. Moreover, the data showed that for individuals with larger DLPFC amplitude, indicative of higher levels of control, the degree of ACC activity was unrelated to behavioral interference. This finding is consistent with the idea that there is reduced need for late-stage selection when the task set is well specified so as to reduce interference from the task-irrelevant processing stream. In contrast, individuals with low DLPFC but high ACC amplitude exhibited a greater degree of interference as measured by the reaction time difference between incongruent and congruent trials, but no more errors than individuals with high DLPFC activity. In contrast, those individuals with both low DLPFC amplitude and low ACC amplitude committed more errors, suggesting that the reduced ability of the ACC to engage in late-stage selection led to compromised performance. An advantage of this approach was that alternative models could be tested. For example, one might argue that this model predicted the data because it posited that the effect of a component occurring earlier in time, that recorded from the DLPFC, was moderated by a component occurring later in time, that recorded from the ACC. Arguing against such an interpretation, a model positing a pathway from an earlier ACC component (in the 220–340 ms time range) via the DLPFC component (at 300–440 ms) did not predict performance. Nor did a model in which activity derived from source location of another brain region involved in cognitive control, RIFG, was substituted for DLPFC (Silton et al., 2010).

Integrating all these findings, we posited a cascade-of-control to control interference in the Stroop task (Banich, 2009). As discussed earlier (and as shown in Figure 1), this model argues that posterior portions of lateral prefrontal cortex are involved in setting a top-down attentional set (i.e., pay attention to ink color) for task-relevant information and act by modulating activity either in one or both of the posterior brain regions that process the task-relevant and task-irrelevant dimension of the Stroop stimulus. This idea is consistent with activation of IFG across distinct meta-analyses of Stroop tasks (Derrfuss et al., 2005). Such task setting can occur even prior to stimulus presentation in a proactive manner (see, for example, Braver, 2012). Once a stimulus appears, relevant information is identified and then mid-DLPFC regions are involved in selecting which of the relevant information should be actively maintained in working memory. Regions of mid-DLPFC have been implicated in buffering relevant information in working memory from interference from competing information (Burgess and Braver, 2010). This information is then sent along to more posterior and dorsal regions of ACC, which are then involved in response-related and late-stage selection, which is required prior to emitting a response. Research with monkeys implicates the ACC as being particularly important for response selection (Isomura et al., 2003). Then, more rostral regions of ACC are involved in response evaluation, which can send a signal back to DLPFC (e.g., Jahn et al., 2014) as posited by the conflict monitoring theory (Botvinick et al., 2004; refer back to Figure 1). Consistent with this notion of a cascade are findings from ERP studies in which the onset of the two stimulus dimensions – task-relevant and task-irrelevant – are varied in time. These studies reveal that ERP waveforms sensitive to stimulus incongruity vary depending on the stimulus onset asynchrony between these two dimensions, implicating a cascading process of interference effects (Appelbaum et al., 2009; Coderre et al., 2011).

## Other Types of Interactions Between Brain Regions that May Influence the Locus of the Stroop Effect

Conceptualizing Stroop interference as occurring via a cascade of control provides additional avenues to consider how the locus of Stroop interference might be considered. In this section, we consider some approaches in that regard. One issue not yet discussed is the mechanism via which top-down biasing by prefrontal regions for a task set influences processing of each of the task-relevant and the task-irrelevant dimension of a Stroop stimulus. One can ask whether interference occurs because the representation of task-relevant information is not adequately upregulated or because the representation of task-irrelevant information is not adequately downregulated. Because of the specificity of brain regions that process each of the two stimulus dimensions contained in Stroop stimuli, one can leverage brain imaging to examine this question.

A number of studies have examined whether, for example, in the standard color-word Stroop task, activity is increased in color processing regions or downregulated in word processing areas (e.g., Egner and Hirsch, 2005; Purmann and Pollmann, 2015). This question is generally approached via the utilization of localizer scans where individuals are shown a series of words and then separately colors to identify, on an individual participant basis, those brain regions that are specifically involved in processing words and then those specifically involved in processing color. One can then examine the degree of activation of each of these regions on average for incongruent trials as compared to congruent trials. Work using such an approach suggests that both mechanisms (upregulation of task-relevant material, downregulation of task-irrelevant material) may occur (e.g., Polk et al., 2008; Coste et al., 2011).

Recently, we have expanded on such approaches to specifically examine how processing of task-relevant vs. task-irrelevant dimensions of a Stroop stimuli predict the degree of Stroop interference that is observed on a trial-by-trial basis (Banich et al., 2019). In our approach, participants performed a localizer task, which in conjunction with multi-voxel pattern analysis (Norman et al., 2006) was used to determine the pattern of brain activity over visual cortex that is specifically associated with processing the task-relevant dimension and then to also determine the pattern of activity associated with the task-irrelevant dimension. The task employed was an emotional word-emotional face Stroop task in which individuals characterized the valence of a word (positive, negative) superimposed on a task-irrelevant emotional face (sad, happy). On each trial, we determined how much activity over posterior cortex was similar to that typical for each dimension (using a classifier fit), that is, how much the pattern of activity looks like face activity and additionally how much the pattern looked like word activity. This approach provided a trial-by-trial readout of how much each dimension was being attended and/or processed.

The important question for purposes of the present article was the degree to which processing of each of these dimensions could predict RT on a given trial and the degree to which such activity occurs as a result of activity in DLPFC modulating activity of posterior brain regions processing each of the task-relevant and task-irrelevant stimulus dimensions. The results yielded different patterns for incongruent as compared to congruent trials. On incongruent trials, greater DLPFC activity directly predicted longer RT, suggesting that when individuals were having difficulty on a given trial, they needed to engage more top-down mechanisms. In addition, more DLFPC activity was associated with less of a classifier fit for faces, suggesting that this brain region is downregulating processing of the task-irrelevant face. However, the degree of processing of the task-relevant face did not predict RT. Hence, interference, at least in the population of individuals in this study, late adolescents, seems to be predicted on incongruent trials by the degree to which DLPFC mechanisms must be engaged. On congruent trials, as on incongruent trials, more DLPFC activity was associated with a poor classifier fit (i.e., less activity) for faces. However, for these trials, more processing of the word was associated with longer RT, suggesting that when more attention needed to be directed to the word to extract the relevant information, RT was elongated.

While these results must be considered in the context that they were obtained in adolescents in whom cognitive control mechanisms are still developing (Andrews-Hanna et al., 2011), they nonetheless raise two important points. First, they provide another example of how brain imaging techniques can be leveraged to try to provide insights into the locus of the Stroop effect that would otherwise be difficult to obtain via behavioral methods alone. Secondly, they suggest that when one talks about the “locus of the Stroop” effect, considered in the context of a cascade, those effects can potentially vary for congruent and incongruent trials, and the interference observed may be a combination of these two effects.

Also suggesting that the locus of the Stroop effect may vary depending on task demands are findings examining the Stroop effect from a network perspective (Spielberg et al., 2015). Using a graph theory approach, higher demand for inhibitory control is associated with restructuring of the global network into a configuration that is more optimized for specialized processing (functional segregation), more efficient at communicating the output of such processing across the network (functional integration), and more resilient to potential interruption (resilience). In addition, there were regional changes with right inferior frontal sulcus and right anterior insula occupying more central positions as network hubs, and dorsal ACC becoming more tightly coupled with its regional subnetwork. This work also suggests that interference is generated via a cascade of activity among regions situated within a larger network and that such configurations can change with control demands on incongruent vs. congruent trials.

## Task-Related Variables that May Influence the Locus of the Stroop Effect

The implications of the results discussed just above, and the model proposed, are that the Stroop effect can occur at a number of different loci and may be influenced by the interaction between these loci as well (e.g., top-down biasing by DLPFC; response-related, late-stage selection by ACC). As a result, it may indeed be that where the Stroop effect is observed is dictated essentially by where your paradigm puts it, even if only implicitly. Two examples are provided here.

First, one of the reasons we used manual responses in most of our fMRI studies was to avoid the potential for head motion that is associated with verbal responding. However, that design choice likely influenced what was observed. In paradigms with a verbal response, there is a much stronger and more automatized mapping between seeing the word (or color) red and verbally producing the word that is associated with it than, for example, training individuals that pressing a button with your index finger denotes “red.” Although we have never formally performed such a comparison, based on prior studies showing differences in activation based on response modality (verbal, manual) during a spatial Stroop task (Barch et al., 2001), one might expect that the interference effects in a vocal color-word Stroop paradigm would more likely involve response-related processing relative to top-down biasing mechanisms, as compared to manual response versions in which there is likely to be less response-related interference. Said differently, pressing an index finger to denote the color red when the word says “blue” is likely to engender less response interference than saying “red” compared to the well-ingrained tendency to say “blue” when seeing the word blue. This idea has been recently supported by a study in which the vocal and manual Stroop effects were compared. The vocal Stroop effect was about twice as large as the manual one. Moreover, ERP recordings indicated that while both the vocal and manual version produced an N400 (suggestive of semantic interference), only for the vocal version was there a response-locked component over left inferior frontal and parietal regions, suggesting additional interference at the level of word production (Zahedi et al., 2019) (however, it should be noted that an alternative suggestion is that different portions of the anterior cingulate are involved in response-related selection for manual vs. vocal tasks; e.g., Liotti et al., 2000; Swick and Turken, 2002).

As a second example, the locus of the Stroop effect may vary depending on the relative automaticity of two processes. One of the reasons that the classic color-word Stroop effect gives such a potent behavioral interference effect is that word reading of color words is so automatic, being some of the earliest learned words. In contrast, the behavioral interference effects for a spatial location–spatial word Stroop task are much less potent. Hence, there may be a greater need for top-down biasing by DLPFC in the former case than the latter.

## Individual Differences that May Influence the Locus of the Stroop Effect

The locus of the Stroop effect may also vary depending on the characteristics of an individual or his/her experience. For example, during the teen years, overcoming interference engendered by Stroop stimuli seem to rely to a greater degree on DLPFC in older adolescents, but on the ACC in younger ones (Andrews-Hanna et al., 2011). Young adults with ADHD appear to show reductions in both DLPFC and ACC activity relative to controls, suggesting disruptions in both top-down and late-stage/response-related aspects of controlling Stroop interference (Banich et al., 2009). Individuals with depression exhibit less DLPFC activity, especially in the left hemisphere (Herrington et al., 2010), with this effect being modulated by level of anxiety (Engels et al., 2010). Moreover, individual differences in approach and avoidance can modulate the lateralization of involvement of the DLPFC in top-down control (Spielberg et al., 2011).

In other individuals, different brain regions other than the typical ones are engaged. For example, women with a history of childhood abuse compared to controls exhibit less fronto-parietal activation, but more activity in regions that are part of the ventral attention/surveillance system during both a standard color-word and color-emotional word Stroop task (Mackiewicz Seghete et al., 2017). In adolescents with severe substance and conduct problems, more activation is observed in medial temporal regions including hippocampal regions (Banich et al., 2007), suggesting potentially a more instance-based processing of Stroop stimuli.

Studies with twins can help to elucidate the potential causes of these effects. For example, in a small sample of monozygotic twins who were discordant for stressful life events, those higher in stressful life events recruited regions of ventrolateral and medial frontal cortex as well as limbic regions while performing an emotional word–emotional face Stroop task. The control co-twins showed only the more typical recruitment of frontoparietal regions thought to be important for executive control of attention and maintenance of task goals. Behavioral performance was not significantly different between twins within pairs, suggesting that the twin who had experienced greater stress recruited additional neural resources associated with affective processing and updating working memory to obtain the same level of behavioral performance (Godinez et al., 2016). A study utilizing a case-control discordant twin pair design revealed that co-twins of individuals with ADHD, like their affected ADHD twin, show reduced activity in the anterior cingulate and insula compared to the unrelated controls, suggesting familial influences. In contrast, portions of the frontoparietal network appear to be the location of effects specific to ADHD, with twins with childhood ADHD showing reduced superior frontal (Brodmann’s Area – BA 6) and parietal region (BA 40) activity compared to both their control co-twins and unrelated control twins (Godinez et al., 2015).

Other work suggests that the nature of the cascade is affected by individual differences. For example, using a source-guided examination of ERP effects, Silton et al. (2011) found that for individuals with high levels of depression, increased LDLPFC activity was directly related to decreased Stroop interference and that ACC did not play an intervening role. Separately for individuals with high levels of anxious apprehension (i.e., worry), higher ACC activity was related to more Stroop interference. These results indicate that depression and anxious apprehension modulate temporally and functionally distinct aspects of the fronto-cingulate network involved in top-down attention control. Additionally, Spielberg et al. (2014) observed that during performance of a color-word Stroop task, increasing levels of anxious arousal were positively associated with coupling of the right DLPFC with orbitofrontal cortex (OFC). In addition, increasing levels of depression were positively associated with right DLPFC–OFC coupling and negatively associated with left DLPFC–OFC coupling. As such, it may be that additional regions to those outlined by our model are brought into the set of regions influencing Stroop interference as a function of individual differences. For example, our model focuses exclusively on cortical regions. Yet at least some research suggests that the ventral tegmental area (VTA)/substantia nigra (SN) and locus coeruleus (LC) also show alterations in activity on incongruent vs. congruent trials, and have differential connectivity to prefrontal regions (Köhler et al., 2016). Hence, individual differences in noradrenergic and/or dopaminergic function may influence the locus of the Stroop effect as well.

## Conclusion

The main takeaway from the work reviewed in this article is that the locus of the Stroop effect can occur at multiple levels from the initiation and creation of a task set for the task goal (e.g., make a decision based on ink color) to late-stage response-related aspects of control. In general, our model suggests that lateral prefrontal regions are more involved in selection and modulation of specific information processing streams (i.e., task-relevant vs. task-irrelevant) while cingulate regions are more involved in late-stage response-related aspects of control. However, even within this general dichotomy, these mechanisms are likely invoked along a cascade, providing the opportunity for control and interference to occur at multiple time points. Additional evidence points to the important role that connectivity between brain regions plays in producing the Stroop effect. Furthermore, the locus of interference may be influenced by the nature of the paradigm (e.g., vocal vs. manual responding in a color-word Stroop task) and by characteristics of an individual.

As such, there is likely no single locus of the Stroop effect, which is both the advantage and the disadvantage of using this task to understand mechanisms of control. On the one hand, if a researcher desires an all-purpose task for examining cognitive control, or alterations to such control, without regard to its locus, the family of Stroop tasks is an excellent choice. One of the reasons we have used Stroop variants in our research is exactly because it is a “broad spectrum” task for detecting deviation in cognitive control. In addition, we chose it because the task instructions are easily understood and, as such, it can be administered across a wide range of ages and with neurologically normal and clinical populations. Moreover, it provides a robust behavioral effect. In addition, while its effect may be more robust at the group than at the individual level (Enkavi et al., 2019), we have found that an interference score [i.e., (Incongruent RT − Congruent RT)/Congruent RT] that accounts for individual differences in overall RT works well especially when combined with neuroimaging. Another aspect of the Stroop task that makes it so versatile is that there are a wide variety of variants that are available.

It is, however, exactly this variation across Stroop paradigms that can be a disadvantage of the task, as it can make comparison across different studies difficult. Researchers often discuss using the “Stroop task” when they use one of the many members of the family of Stroop task variants. Yet, each variant of the task likely generates the need for control at different loci, as our research has demonstrated. To help facilitate comparison across studies, we have tried to be more explicit in our task nomenclature by indicating both the task-relevant and task-irrelevant dimensions (e.g., the classic color-word Stroop task). If this nomenclature were adopted more broadly across the field, it might facilitate comparisons across studies. However, to truly facilitate comparison, it would also be important to indicate the nature of neutral trials. In some studies of the classic color-word Stroop task, the neutral trials are simply a series of colored “xxxxxxx”s. Such stimuli are not as likely to engage word reading mechanisms as, for example, the neutral non-color words that we have typically employed (refer back to discussion in section “Interference Between Two Processes That Vary in Their Automaticity or Control Demands”), which will also influence the locus of the Stroop effect (as will the specific contrast being examined, e.g., incongruent vs. neutral trials, incongruent vs. congruent trials).

In conclusion, the “Stroop task” can be used either more as a hammer to detect cognitive control across a variety of loci in a broad-based manner or more as a scalpel to investigate control at a very limited level if designed with specifically constrained stimuli and contrasts. Just as there is a family of Stroop tasks, there is also a family of loci at which the Stroop effect can occur. Moreover, the different loci may be generated across a series of distinct but interacting brain regions to produce the single behavioral effect that is observed.

## Author Contributions

The author confirms being the sole contributor of this work and has approved it for publication.

## Conflict of Interest

The author declares that the research was conducted in the absence of any commercial or financial relationships that could be construed as a potential conflict of interest.
